# Genome sequence of a divergent strain of canine distemper virus detected in New Zealand fur seals

**DOI:** 10.1128/mra.00151-25

**Published:** 2025-04-28

**Authors:** Andrew Wilson, Edna Gias, Alvey Little, Ruy Jauregui, Yee Syuen Low, David Pulford, Angela Steyn, Keanan Sylvester, Daniel Green, Joseph O'Keefe, Michelle McCulley

**Affiliations:** 1 Animal Health Laboratory, Diagnostics, Readiness and Surveillance, Biosecurity New Zealand, Ministry for Primary Industries, Upper Hutt, New Zealand; Katholieke Universiteit Leuven, Leuven, Belgium

**Keywords:** morbillivirus, metagenomics, New Zealand

## Abstract

We report the draft genome sequence of a strain of canine distemper virus detected in New Zealand fur seals (*Arctocephalus forsteri*). A high-quality draft consensus genome was produced through a combination of long- and short-read metagenomic sequencing approaches. Phylogenetic analysis confirms that the virus is a divergent strain of canine distemper virus.

## ANNOUNCEMENT

Canine distemper viruses (CDVs) are single-stranded RNA viruses, belonging to the genus *Morbillivirus* in the family *Paramyxoviridae,* which cause pantropic infection (1). Although canines are the most common host, CDV can affect multiple domestic and wild mammalian species, including ferrets, felines, and pinnipeds (2).

In late 2024, a mortality event involving New Zealand fur seals (*Arctocephalus forsteri*) was reported on the South Island of New Zealand. Swabs (*n* = 8) and tissue samples (*n* = 8) of the brain, lung, and trachea from multiple seals were delivered to the New Zealand Ministry for Primary Industries Animal Health Laboratory for exotic disease investigation (accession W24_02531). Tissue samples were manually homogenized in a BIOREBA extraction bag (BIOREBA AG), then RNA was extracted using the QIAamp Viral RNA Kit (Qiagen). All samples (*n* = 16) tested positive for canine and phocine morbilliviruses using a pan-genotypic RT-qPCR assay, with lung tissue producing the strongest positive signal (3). RNA from the lung was converted to cDNA using the Maxima H Minus Double-Stranded cDNA Synthesis Kit (Thermo Fisher Scientific).

Libraries for long-read metagenomics were prepared using the Rapid PCR Barcoding Kit V14 (SQK-RPB114.24, Oxford Nanopore Technologies [ONT]) and sequenced on a MinION Mk1B with a R10.4.1 flow cell (ONT). Basecalling was performed with Dorado version 7.4.14 using the high-accuracy model version 4.3.0. In total, 18,202 reads were generated with an *N*_50_ of 5.37 Kb. Raw reads were trimmed using BBDuk version 38 (4), then mapped to reference canine and phocine morbillivirus genomes (NCBI references MW713449.1 and KC802221.1) using Minimap2 version 2.24 (5).

For short-read metagenomic sequencing, libraries were prepared using the Nextera XT kit (Illumina). Sequencing was performed on a MiSeq (Illumina), using a 500-cycle (2 × 250 bp) reagent kit v2. Raw reads were trimmed using BBDuk version 38.84 (4), then mapped to the long-read consensus sequence using BBMap version 38.84 (4) in Geneious Prime version 2021.1.1 (https://www.geneious.com). A hybrid consensus genome sequence was then generated using standard functions in Geneious Prime.

Long-read mapping to MW713449.1 produced a higher quality draft genome compared to mapping to KC802221.1 (10× coverage) and was therefore used as a reference sequence for short-read mapping. After mapping short reads to the long-read consensus sequence, a coding-complete genome sequence was generated. Genome assembly statistics for the hybrid consensus genome are provided in Table 1.

**TABLE 1:** Genome assembly statistics and accession numbers

Feature	W24_02531 hybrid consensus genome (short reads mapped to long-read consensus genome)
Closest reference accession (pairwise identity, %)	JN896987.1 (87.48%)
Raw read number	4,931,444
Number of mapped reads	1,002,224 (20.3%)
Assembled genome size (bp)	15,392
Bases called at or above Q30	>99.9%
Mean depth of coverage	15,303×
Number of contigs	1
GC content	42.4%
Completeness	97.5%, coding complete

Open reading frames (ORFs) were predicted using the “Find ORFs” function in Geneious Prime. Genes were annotated using various morbillivirus reference genomes obtained from GenBank. Genome completeness was estimated using CheckV version 1.0.1 (6). Alignments were generated using MAFFT version 7.490 (7). Phylogenetic trees were constructed in MEGA11 (8).

All laboratory protocols detailed above were performed according to the manufacturer’s instructions. For software, default parameters were used.

**Fig 1:**
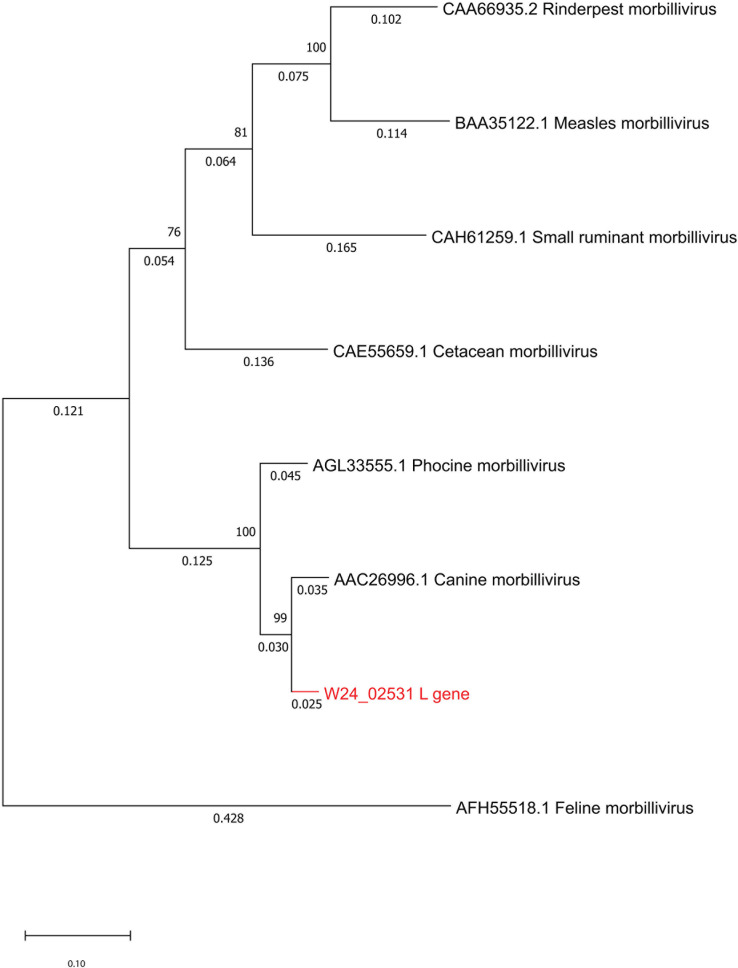
Phylogenetic analysis of the L protein amino acid sequences of the genus *Morbillivirus* and the genome reported here (in red). Complete L protein amino acid sequences were aligned using MAFFT version 7.490. Evolutionary history was inferred by using the maximum likelihood method and the Jones-Taylor-Thornton (JTT) matrix-based model. The tree with the highest log likelihood (−20,522.17) is shown. The percentage of 500 trees in which the associated taxa clustered together is shown next to the branches. Initial trees for the heuristic search were obtained automatically by applying Neighbor-Join and BioNJ algorithms to a matrix of pairwise distances estimated using the JTT model and then selecting the topology with the superior log likelihood value. The tree is drawn to scale, with branch lengths measured in the number of substitutions per site (next to the branches). This analysis involved eight amino acid sequences. There were a total of 2,751 positions in the final data set. Evolutionary analyses were conducted in MEGA11.

The International Committee on Taxonomy of Viruses demarcates *Morbillivirus* species based on translated L gene segment comparison and branch length estimation in a phylogenetic tree. To be classified as a new species, branch length must be ≥0.03 (9). The reported genome has a branch length of 0.025 and falls within the *Morbillivirus canis* clade (Fig. 1), classifying it as a divergent strain of CDV.

## Data Availability

Raw sequence data have been submitted to NCBI’s Sequence Read Archive (SRA) under BioProject accession number PRJNA1222009. The genome reported here has been deposited in GenBank under accession PV102958.
